# CTLA-4 Expression Inversely Correlates with Kidney Function and Serum Immunoglobulin Concentration in Patients with Primary Glomerulonephritides

**DOI:** 10.1007/s00005-019-00548-3

**Published:** 2019-06-08

**Authors:** Ewelina Grywalska, Iwona Smarz-Widelska, Sebastian Mertowski, Krzysztof Gosik, Michał Mielnik, Martyna Podgajna, Monika Abramiuk, Bartłomiej Drop, Jacek Roliński, Wojciech Załuska

**Affiliations:** 1 Department of Clinical Immunology and Immunotherapy, Medical University of Lublin, Chodzki 4a, 20-093 Lublin, Poland; 2 Department of Nephrology, Cardinal Stefan Wyszynski Provincial Hospital in Lublin, Lublin, Poland; 3 Department of Hematooncology and Bone Marrow Transplantation, Medical University of Lublin, Lublin, Poland; 4 The First Department of Gynecologic Oncology and Gynecology, Medical University of Lublin, Lublin, Poland; 5 Department of Informatics and Medical Statistics, Medical University of Lublin, Lublin, Poland; 6 Department of Nephrology, Medical University of Lublin, Lublin, Poland

**Keywords:** Cytotoxic T-lymphocyte-associated antigen-4, Glomerulonephritides, kidney function

## Abstract

Major causes of chronic kidney disease are primary proliferative and nonproliferative glomerulonephritides (PGN and NPGN). However, the pathogenesis of PGN and NPGN is still not fully understood. Cytotoxic T-lymphocyte-associated antigen-4 (CTLA-4) is a T-cell membrane receptor that plays a key role in T-cell inhibition. Despite its role in autoimmunological diseases, little is known about the involvement of CTLA-4 in the pathogenesis of PGN and NPGN. The objective of this study was to determine the role of CTLA-4 in the pathogenesis of PGN and NPGN by evaluating the frequencies of T and B lymphocytes expressing CTLA-4 and the serum concentration of the sCTLA-4 isoform in patients with PGN and NPGN in relation to clinical parameters. The study included peripheral blood (PB) samples from 40 PGN and NPGN patients and 20 healthy age- and sex-matched volunteers (control group). The viable PB lymphocytes were labeled with fluorochrome-conjugated monoclonal anti-CTLA-4 antibodies and analyzed using flow cytometry. The serum concentration of sCTLA-4 was measured using ELISA. The frequencies and absolute counts of CD4^+^/CTLA-4^+^ T lymphocytes, CD8^+^/CTLA-4^+^ T lymphocytes and CD19^+^/CTLA-4^+^ B lymphocytes and the serum sCTLA-4 concentration were lower in PGN and NPGN patients that in the control group. Reduced sCTLA-4 expression was associated with a lower concentration of serum immunoglobulins. Our results indicate that deregulation of CTLA-4 expression may result in continuous activation of T cells and contribute to the pathogenesis of PGN and NPGN.

## Introduction

Chronic kidney disease affects 13.4% of the population worldwide (Hill et al. 2016), leading to millions of deaths each year (Krata et al. 2018). Major causes of chronic kidney disease and end-stage renal disease (MD 2013) are primary glomerulonephritides (GN). Primary GN are categorized into proliferative GN (PGN), characterized by an increased number of cells in glomeruli, and nonproliferative GN (NPGN), characterized by a lack of proliferation of cells in glomeruli. Primary GN are a heterogeneous group of glomerular diseases characterized by local inflammation of glomeruli. Local glomerular inflammation is caused by dysregulated humoral and cellular immune responses to different etiologic agents (Floege 2013; Rodrigues et al. 2014). During humoral response, B cells are activated, immunoglobulin (Ig) deposited, and complement activated. During cellular response, circulating mononuclear inflammatory cells (including lymphocytes and macrophages) are infiltrated into glomeruli and lead to crescent formation (Krebs and Steinmetz 2016). However, the underlying molecular mechanism and pathogenesis of PGN and NPGN are still not fully understood.

Cytotoxic T-lymphocyte-associated antigen-4 (CTLA-4) is a member of the immunoglobulin superfamily (Brunet et al. 1987) that inhibits the activation of T cells (Walunas et al. 1994) and plays a key role in the priming phase of the immune response (Scalapino and Daikh 2008). Although CTLA-4 was initially identified as a T-cell-specific protein, it is now known to be expressed in B cells (Quandt et al. 2007) and dendritic cells (Mackern-Oberti et al. 2017) also. CTLA-4 inhibits T-cell responses by several mechanisms. In resting T cells, CTLA-4 is found in the intracellular compartment. After T-cell activation through CD28 binding, CTLA-4 is transported to the membrane and expressed on the surface of T cells (Linsley et al. 1996; Walunas et al. 1994). Once on the T-cell surface, CTLA-4 binds B7-1 (CD80) and B7-2 (CD86) ligands on activated antigen-presenting cells (B cells, monocytes) with a 100-fold higher affinity than CD28. As a result, further costimulation of the T-cell receptor–major histocompatibility complex bundle by CD28-B7 is inhibited (Krummel and Allison 1995). Moreover, CTLA-4 can inhibit T-cell activation by eliciting direct signal transduction, resulting, for example, in the activation of phosphoinositide 3-kinase. CTLA-4 can also induce an inhibitory phenotype in dendritic cells by retrograde signaling via B7.1/B7.2 on dendritic cells (Grohmann et al. 2002; Hoff et al. 2009, 2010). The complexity of the CTLA-4 function is further increased by the presence of several alternatively spliced variants. Apart from the full-length membrane form of CTLA-4 that has a ligand-binding extracellular domain, a transmembrane domain, and an intracellular signaling domain, three other isoforms exist: sCTLA-4, liCTLA-4 and 1/4CTLA-4. The soluble isoform sCTLA-4 is expressed by non-stimulated T cells and, despite lacking a transmembrane domain, can bind CD80/CD86 (Magistrelli et al. 1999; Oaks et al. 2000). The ligand-independent isoform liCTLA-4 is expressed in naive and activated T cells and, due to the lack of a B7 binding domain, is unable to bind CD80/CD86 (Vijayakrishnan et al. 2004). The short variant 1/4CTLA-4 lacks both the ligand-binding domain and the transmembrane domain (Ueda et al. 2003). Due to single nucleotide polymorphisms (SNP) in the CTLA-4 gene, regulation of the amount of the different splice variants is altered, leading to an increased susceptibility to several autoimmune diseases (Ueda et al. 2003).

Despite the important role CTLA-4 plays in the pathogenesis of autoimmunological (Holmberg et al. 2005) and cancer diseases (Ghaderi 2011), little is known about its involvement in the pathogenesis and progression of primary GN.

The objective of this study was to evaluate the role of CTLA-4 in the pathogenesis of primary GN. We analyzed the frequencies of T and B lymphocytes expressing CTLA-4 in peripheral blood (PB) and the plasma concentration of the sCTLA-4 isoform in patients with PGN and NPGN in relation to clinical parameters.

## Materials and Methods

### Patients and Healthy Volunteers

In this study, we analyzed PB samples from 40 randomly selected, newly diagnosed, previously untreated patients (24 men and 16 women) with primary GN and 20 healthy age- and sex-matched healthy volunteers (control group). Twelve patients were diagnosed with PGN: nine patients with IgA nephropathy (four women, five men) and three patients with membranoproliferative glomerulonephritis (two women, one man). Twenty-eight patients were diagnosed with NPGN: 16 patients with minimal change disease (MCD; seven women, nine men), eight patients with membranous glomerulonephritis (two women, six men), and four patients with focal segmental glomerulosclerosis (two women, two men). Neither the patients nor the controls used immunomodulating agents or hormonal preparations, underwent blood transfusion, showed signs of infection within at least 3 months prior to the study, or presented with autoimmune conditions or allergies. Moreover, the controls did not have a history of oncological therapy, prior treatment for tuberculosis, or other chronic conditions that could be related to impaired humoral or cellular immunity. Primary GN was diagnosed on the basis of standard diagnostic criteria, with special emphasis on kidney biopsy (Floege and Amann 2016).

This study was approved by the Ethics Committee of the Medical University of Lublin (Decision No. KE-0254/290/2014). Written informed consent was obtained from all patients regarding the use of their blood for scientific purposes. This study was carried out according to the Declaration of Helsinki.

### Isolation of PB Mononuclear Cells

PB mononuclear cells were isolated from patients with PGN and NPGN and healthy controls. Venous blood samples (5 mL) were collected by venipuncture using sterile, lithium heparin-treated tubes (S-Monovette, SARSTEDT, Aktiengesellschaft & Co., Numbrecht, Germany). Standard density gradient centrifugation was used (Gradisol L, Aqua Medica, Poland) for the aseptic separation of PB mononuclear cells.

### Flow Cytometry

The percentages of cells expressing surface markers were analyzed using flow cytometry. The cells were incubated for 20 min, in the dark and at room temperature, with a combination of fluorochrome-labeled [fluorescein isothiocyanate (FITC), phycoerythrin (PE),CyChrome (Cy5)] mouse anti-human monoclonal antibodies (mAbs): CD45 FITC/CD14 PE, CD3 PE, CD19 FITC, CD4 FITC, CD8 FITC, CTLA-4 PE-Cy5 (BD Biosciences, San Jose, CA, USA). For three-color immunofluorescence analyses, a FACSCalibur flow cytometer (Becton-Dickinson, Franklin Lakes, NJ, USA) equipped with a 488-nm argon laser was used. At least 10,000 events were acquired and analyzed using CellQuest Software. The FACS data were evaluated using dot plots. Exemplary data for PGN, NPGN and control patients are shown in S1, S2 and S3, respectively. Analyzed cells were determined by forward and side scattering, single color vs. side scatter and two-color fluorescence plot. The relative proportion of cells expressing surface markers was quantified by placing gates around the distinct populations. The results are presented as the percentage of CD45^+^ cells. The percentage of positive cells was calculated by comparing with the control. Isotype-matched, directly conjugated FITC mouse IgG1 κ isotype control and PE mouse IgG1 κ isotype control mAbs were used for background determination. To exclude debris and cell aggregates, the samples were gated on forward scatter vs. side scatter.

### ELISA

The commercial enzyme-linked immunosorbent assay (ELISA) kit CTLA-4 (Soluble) Human ELISA Kit (Thermo Fisher Scientific, Waltham, MA, USA), with a sensitivity of 0.13 ng/mL and assay range 0.16–10.0 ng/mL, was used for the quantitative determination of human sCTLA-4 in plasma samples. For plasma separation, 5-mL samples of PB collected into EDTA tubes were used. Plasma samples were stored in liquid nitrogen until the time of analysis. The analysis was performed in accordance with the manufacturer’s recommendations. The ELISA Reader VictorTM3 (PerkinElmer, Waltham, MA, USA) microplate reader was used for measurements.

### Statistical Analysis

The Shapiro–Wilk test was used to test the normality of continuous variables. The statistical characteristics of continuous variables are presented as medians, minimum and maximum values, and arithmetic means and their standard deviations (SD). Student’s *t* test was used for comparisons between independent variables, and the Mann–Whitney *U* test was used for intergroup comparisons. The power and direction of relationships between pairs of continuous variables were determined using Spearman’s coefficient of rank correlation. The distributions of discrete variables were compared with Pearson’s Chi-square test or Fisher’s exact test. Receiver operating characteristic (ROC) curves were generated for significant predictor variables of PGN patients. The following variables were included: frequencies of CD4^+^/CTLA-4^+^ T lymphocytes (among CD4^+^ T lymphocytes); CD4^+^/CTLA-4^+^ T lymphocytes (among CD4^+^ T lymphocytes) (10^3^/mm^3^]; frequencies of CD8^+^/CTLA-4^+^ T lymphocytes (among CD8^+^ T lymphocytes) (%]; CD8^+^/CTLA-4^+^ T lymphocytes (among CD8^+^ T lymphocytes) (10^3^/mm^3^); frequencies of CD19^+^/CTLA-4^+^ B lymphocytes (among CD19^+^ B lymphocytes) (%);CD19^+^/CTLA-4^+^ B lymphocytes (among CD19^+^ B lymphocytes) (10^3^/mm^3^); and serum CTLA-4 concentration [ng/mL]. The areas under the ROC curves were calculated and compared for each parameter. All the calculations were carried out with the Statistica 10 (StatSoft^®^, Palo Alto, CA, USA) package and Graphpad Prism 5 (GraphPad Software, San Diego, CA, USA). Differences were considered statistically significant with a *p *< 0.05.

## Results

### Characteristics of Patients and Controls

The levels of selected proteins, complement components, and renal function parameters of the patients and controls are presented in Table 1. Their complete blood count and basic lymphocyte subsets are presented in Table 2.

**Table 1:** Levels of selected proteins, complement components, and renal function parameters in PGN patients, NPGN patients, and control group

Parameters	PGN		NPGN		Control group		Group of patients		
							PGN vs. NPGN	PGN vs. control	NPGN vs. control
	Mean ± SD	Median (range)	Mean ± SD	Median (range)	Mean ± SD	Median (range)	*p* value	*p* value	*p* value
Age (years)	43.58 ± 11.01	41.50 (28.00–65.00)	42.89 ± 17.41	39.50 (19.00–75.00)	44.00 ± 12.22	45.00 (20.00–61.00)	> 0.9999	> 0.9999	> 0.9999
Urea (mg/dL)	36.84 ± 13.55	36.77 (13.03–54.76)	50.68 ± 29.65	37.98 (17.79–115.7)	31.4 ± 6.946	32.00 (18.00–42.00)	> 0.9999	> 0.9999	0.1465
BUN (mg/dL)	17.21 ± 6.33	17.18 (6.089–25.59)	23.68 ± 13.85	17.75 (8.313–54.05)	14.67 ± 3.246	14.95 (8.411–19.63)	> 0.9999	> 0.9999	0.1465
Serum creatinine (mg/dL)	1.065 ± 0.381	1.035 (0.54–1.79)	1.101 ± 0.5505	0.9 (0.37–2.3)	0.9235 ± 0.1225	0.925 (0.7–1.13)	> 0.9999	> 0.9999	> 0.9999
Serum uric acid (mg/dL)	7.083 ± 1.472	7.75 (4.00–8.40)	6.354 ± 1.887	5.85 (3.8–11.9)	6.22 ± 1.401	6.95 (3.7–7.9)	0.2944	0.2390	> 0.9999
Serum IgG (g/L)	7.234 ± 2.264	7.35 (3.42–10.84)	4.556 ± 2.291	4.56 (2.00–13.25)	12.71 ± 1.403	12.79 (10.06–15.47)	0.1047	0.0050	< 0.0001
Serum IgM (g/L)	1.46 ± 0.7495	1.2 (0.5–3.2)	1.316 ± 0.8221	1.05 (0.4–3.0)	1.664 ± 0.3139	1.61 (1.17–2.19)	> 0.9999	0.4636	0.0223
Serum IgA (g/L)	2.891 ± 1.389	3.235 (0.77–4.67)	2.251 ± 1.111	2.335 (0.6–5.69)	2.386 ± 0.8379	2.56 (0.92–3.92)	0.3064	0.8786	> 0.9999
Serum total protein (g/dL)	5.575 ± 0.9006	5.65 (4.2–7.1)	4.818 ± 1.052	4.8 (3.2–7.4)	7.354 ± 0.5757	7.35 (6.4–8.2)	0.3302	0.0024	< 0.0001
Serum albumin (g/L)	3.155 ± 0.6313	3.2 (2.1–3.9)	2.337 ± 0.8215	2.3 (0.6–3.8)	4.18 ± 0.357	4.225 (3.5–4.75)	0.1132	0.0058	< 0.0001
Total protein in a 24-h urine collection test (g/24 h)	3.025 ± 2.179	2.12 (0.4–7.03)	6.524 ± 5.452	4.815 (1.00–20.00)	0.0 ± 0.0	0.0	0.3933	0.0004	< 0.0001
Serum complement component C3 (g/L)	1.15 ± 0.2748	1.2 (0.4–1.5)	1.347 ± 0.2893	1.3 (0.9–2.0)	1.284 ± 0.2158	1.235 (0.95–1.78)	0.4181	> 0.9999	> 0.9999
Serum complement component C4 (g/L)	0.2543 ± 0.05114	0.2695 (0.11–0.32)	0.3192 ± 0.09133	0.3 (0.2–0.55)	0.2825 ± 0.07725	0.285 (0.15–0.39)	0.2544	> 0.9999	0.9755

*BUN* blood urea nitrogen, *NPGN* nonproliferative glomerulonephritis, *PGN* proliferative glomerulonephritis

**Table 2:** Complete blood count and basic lymphocyte subsets in patients with PGN, NPGN, and healthy volunteers

Parameters	PGN		NPGN		Control group		Group of patients		
							PGN vs. NPGN	PGN vs. control group	NPGN vs. control group
	Mean ± SD	Median (range)	Mean ± SD	Median (range)	Mean ± SD	Median (range)	*p* value	*p* value	*p* value
WBC (10^3^/mm^3^)	7.567 ± 1.867	7.215 (4.3–9.94)	6.506 ± 1.567	6.295 (4.38–9.8)	6.821 ± 0.4158	6.72 (6.26–7.61)	0.0821	> 0.9999	0.4620
LYM (10^3^/mm^3^)	2.138 ± 0.8505	1.855 (1.2–3.74)	2.055 ± 0.6686	1.930 (1.2–3.7)	2.499 ± 0.5592	2.535 (1.53-3.7)	> 0.9999	0.2390	0.0678
RBC [10^6^/mm^3^]	4.362 ± 0.5362	4.38 (3.34–5.1)	4.646 ± 1.468	4.4 (3.66–11.8)	5.171 ± 0.4252	5.12 (4.5–5.8)	> 0.9999	0.0011	< 0.0001
HGB [g/dL]	12.88 ± 1.717	13.1 (9.3–15.1)	13.36 ± 1.535	13.3 (10.7–16.4)	14.3 ± 1.189	14.35 (12.5–16.9)	> 0.9999	0.0686	0.1056
PLT (10^3^/mm^3^)	242.1 ± 77.78	212.5 (177.0–410.0)	239.8 ± 58.67	235.5 (120.0–361.0)	279.0 ± 57.05	281.5 (186.0–403.0)	> 0.9999	0.1389	0.1257
CD3^+^ T lymphocytes (%)	71.65 ± 4.398	72.9 (59.72–75.38)	73.82 ± 8.197	73.29 (49.83–85.14)	68.26 ± 3.842	68.08 (60.63–74.49)	> 0.9999	0.0946	0.0010
CD3^+^ T lymphocytes (10^3^/mm^3^)	1.54 ± 0.65	1.359 (0.8436–2.796)	1.531 ± 0.5612	1.487 (0.6478–2.970)	1.697 ± 0.3418	1.724 (1.067–2.243)	> 0.9999	0.4770	0.3932
CD19^+^ B lymphocytes (%)	12.28 ± 4.495	13.6 (5.49–18.91)	10.75 ± 5.078	10.74 (3.96–20.73)	11.25 ± 2.502	11.4 (6.04–16.9)	0.6972	> 0.9999	> 0.9999
CD19^+^ B lymphocytes (10^3^/mm^3^)	0.2653 ± 0.1388	0.2587 (0.09333–0.4671)	0.23 ± 0.1512	0.1733 (0.06384–0.5654)	0.2852 ± 0.09876	0.296 (0.1166–0.4631)	0.9786	> 0.9999	0.1551
CD3^+^/CD4^+^ T lymphocytes (%)	41.59 ± 8.358	43.58 (26.13–50.75)	40.29 ± 8.131	39.23 (25.69–54.97)	44.46 ± 2.505	44.16 (40.71–48.84)	> 0.9999	> 0.9999	0.2255
CD3^+^/CD4^+^ T lymphocytes (10^3^/mm^3^)	0.653 ± 0.3183	0.6464 (0.277–1.285)	0.6141 ± 0.2709	0.5631 (0.2457–1.598)	0.7579 ± 0.1743	0.7655 (0.4576–1.071)	> 0.9999	0.3889	0.0331
CD3^+^/CD8^+^ T lymphocytes (%)	25.77 ± 5.528	26.72 (18.13–34.59)	30.18 ± 8.721	29.38 (14.82–54.00)	34.36 ± 3.286	34.74 (29.33–39.6)	0.2626	0.0008	0.0325
CD3^+^/CD8^+^ T lymphocytes (10^3^/mm^3^)	0.396 ± 0.2069	0.3372 (0.2174–0.9625)	0.481 ± 0.2696	0.4275 (0.1153–1.425)	0.5799 ± 0.1127	0.5859 (0.3697–0.7803)	0.5168	0.0046	0.0567
T CD3^+^/CD4^+^: T CD3^+^/CD8^+^ lymphocyte ratio	1.736 ± 0.6687	1.685 (0.85–2.74)	1.491 ± 0.5974	1.48 (0.4757–2.632)	1.307 ± 0.1571	1.291 (1.028–1.569)	0.8162	0.3008	> 0.9999

*HGB* hemoglobin, *LYM* lymphocytes, *NPGN* nonproliferative glomerulonephritis, *PGN* proliferative glomerulonephritis, *PLT* platelets, *RBC* red blood cells, *WBC* white blood cells

### Frequencies and Absolute Counts of CTLA-4^+^ Lymphocytes are Lower in Patients with PGN and NPGN than in Controls

The frequencies and absolute counts of CTLA-4^+^ T and B lymphocytes detected by flow cytometry in the PB mononuclear cell samples of patients and controls are presented in Table 3.

**Table 3:** Frequencies and absolute counts of CTLA-4- + lymphocytes and serum sCTLA-4 concentration in patients with PGN, NPGN, and healthy volunteers

Parameters	PGN		NPGN		Control group		Group of patients		
							PGN vs. NPGN	PGN vs. control group	NPGN vs. control group
	Mean ± SD	Median (range)	Mean ± SD	Median (range)	Mean ± SD	Median (range)	*p* value	*p* value	*p* value
CD4^+^/CTLA-4^+^ T lymphocytes (among CD4^+^ T lymphocytes) (%)	1.549 ± 1.327	1.265 (0.11–3.96)	1.886 ± 1.397	1.905 (0.02–5.45)	6.793 ± 1.280	6.665 (4.37–9.16)	> 0.9999	< 0.0001	< 0.0001
CD4^+^/CTLA-4^+^ T lymphocytes (among CD4^+^ T lymphocytes) (10^3^/mm^3^)	0.0106 ± 0.0095	0.0079 (0.0003–0.0293)	0.0099 ± 0.0078	0.0095 (0.0002–0.0352)	0.0510 ± 0.0132	0.0470 (0.0267–0.0757)	> 0.9999	< 0.0001	< 0.0001
CD8^+^/CTLA-4^+^ T lymphocytes (among CD8^+^ T lymphocytes) (%)	2.033 ± 1.079	1.995 (0.58–4.05)	1.254 ± 1.144	1.03 (0.05–4.6)	5.176 ± 1.779	5.445 (1.77–7.99)	0.3503	0.0037	< 0.0001
CD8^+^/CTLA-4^+^ T lymphocytes (among CD8^+^ T lymphocytes) (10^3^/mm^3^)	0.0083 ± 0.0058	0.0077 (0.0016–0.0195)	0.0056 ± 0.0054	0.0035 (0.0002–0.0191)	0.0296 ± 0.0110	0.0295 (0.0105–0.0525)	0.8213	0.0005	< 0.0001
CD19^+^/CTLA-4^+^ B lymphocytes (among CD19^+^ B lymphocytes) (%)	0.6533 ± 1.211	0.3 (0.13–4.48)	2.172 ± 1.029	2.295 (0.53–4.14)	5.435 ± 2.118	5.68 (2.39–9.48)	0.0085	< 0.0001	0.0002
CD19^+^/CTLA-4^+^ B lymphocytes (among CD19^+^ B lymphocytes) (10^3^/mm^3^)	0.0011 ± 0.0010	0.0010 (0.0002–0.0042)	0.0044 ± 0.0027	0.0035 (0.0007–0.0115)	0.0153 ± 0.0077	0.0146 (0.0042–0.0303)	0.0080	< 0.0001	< 0.0001
Serum CTLA-4 concentration [ng/mL]	2.262 ± 1.511	2.303 (0.179–5.485)	3.14 ± 1.489	3.12 (0.769–7.94)	6.349 ± 1.364	6.12 (4.603–9.648)	0.7291	< 0.0001	< 0.0001

*NPGN* nonproliferative glomerulonephritis, *PGN* proliferative glomerulonephritis

**Fig. 1:**
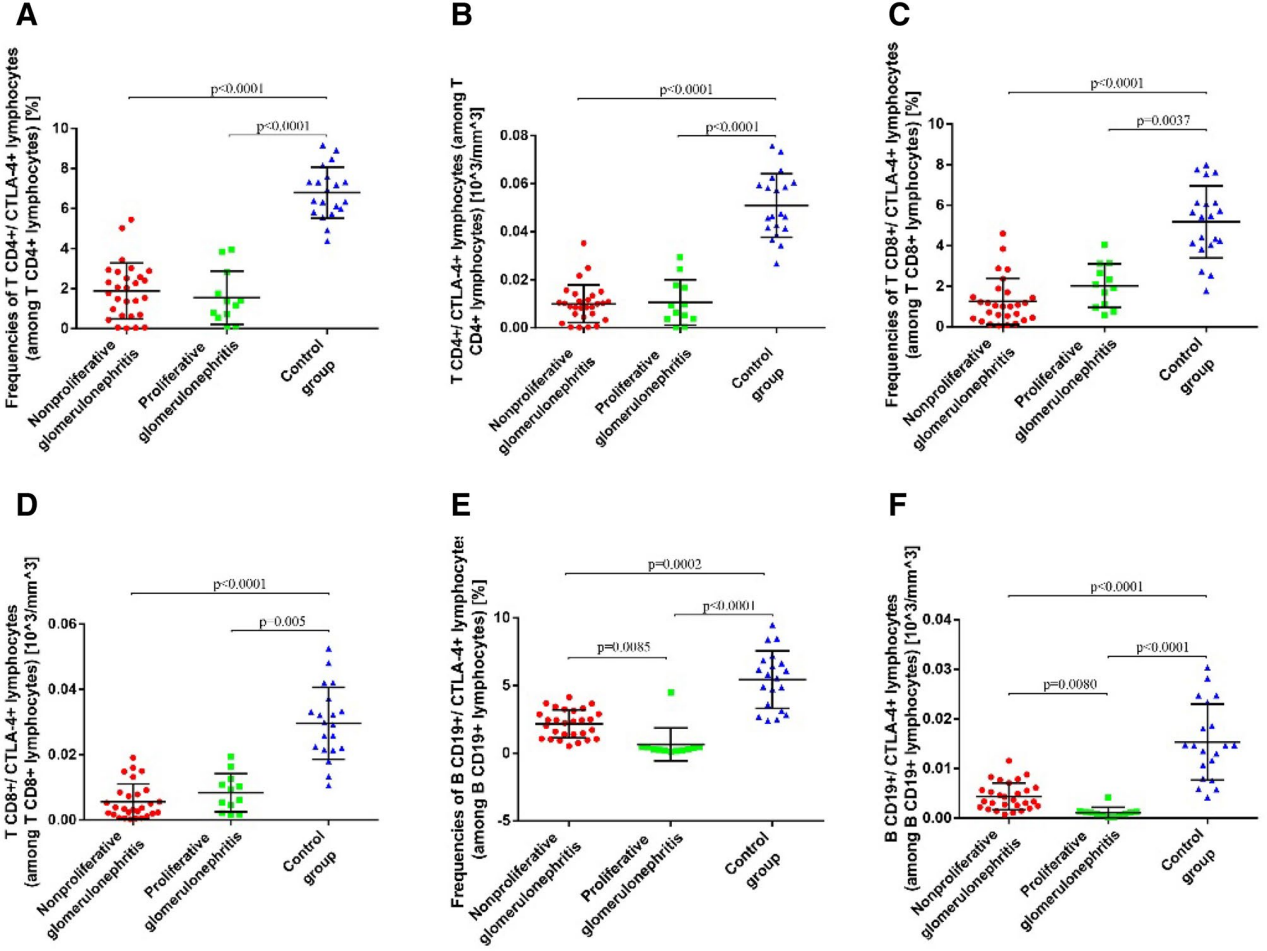
**a** Frequencies of CD4^+^/CTLA-4^+^ T lymphocytes in patients with proliferative glomerulonephritis, patients with nonproliferative glomerulonephritis, and healthy volunteers. **b** The absolute counts of CD4^+^/CTLA-4^+^ T lymphocytes (among CD4^+^ T lymphocytes) in patients with proliferative glomerulonephritis, patients with nonproliferative glomerulonephritis, and healthy volunteers. **c** The frequencies of CD8^+^/CTLA-4^+^ T lymphocytes in patients with proliferative glomerulonephritis, patients with nonproliferative glomerulonephritis, and healthy volunteers. **d** The absolute counts of CD8^+^/CTLA-4^+^ T lymphocytes (among CD8^+^ T lymphocytes) in patients with proliferative glomerulonephritis, patients with nonproliferative glomerulonephritis, and healthy volunteers. **e** The frequencies of CD19^+^/CTLA-4^+^ B lymphocytes in patients with proliferative glomerulonephritis, patients with nonproliferative glomerulonephritis, and healthy volunteers. **f** The absolute counts of CD19^+^/CTLA-4^+^ B lymphocytes (among CD19^+^ B lymphocytes) in patients with proliferative glomerulonephritis, patients with nonproliferative glomerulonephritis, and healthy volunteers

The frequencies of CD4^+^/CTLA-4^+^ T lymphocytes were lower among patients with PGN [1.549 ± 1.327%; median 1.265% (0.11–3.96%)] and NPGN [1.886 ± 1.397%; median 1.905% (0.02–5.45%)] than in the control group [6.793 ± 1.280%; median 6.665% (4.37–9.16%)] (Fig. 1a). The corresponding absolute count graph is presented in Fig. 1b.

The frequencies of CD8^+^/CTLA-4^+^ T lymphocytes were also lower among patients with PGN [2.033 ± 1.079%; median 1.995% (0.58–4.05%)] and NPGN [1.254 ± 1.144%; median 1.03% (0.05–4.6%)] than in the control group [5.176 ± 1.779%; median 5.445% (1.77–7.99%)] (Fig. 1c). The corresponding absolute count graph is presented in Fig. 1d.

Similarly, the frequencies of CD19^+^/CTLA-4^+^ B lymphocytes were lower among patients with PGN [0.6533 ± 1.211%; median 0.3% (0.13–4.48%)] and NPGN [2.172 ± 1.029%; median 2.295% (0.53–4.14%)] than in the control group [5.435 ± 2.118%; median 5.68% (2.39–9.48%)] (Fig. 1e). The corresponding absolute count graph is presented in Fig. 1f.

### Serum sCTLA-4 Concentration is Lower in Patients with PGN and NPGN than in Controls

**Fig. 2:**
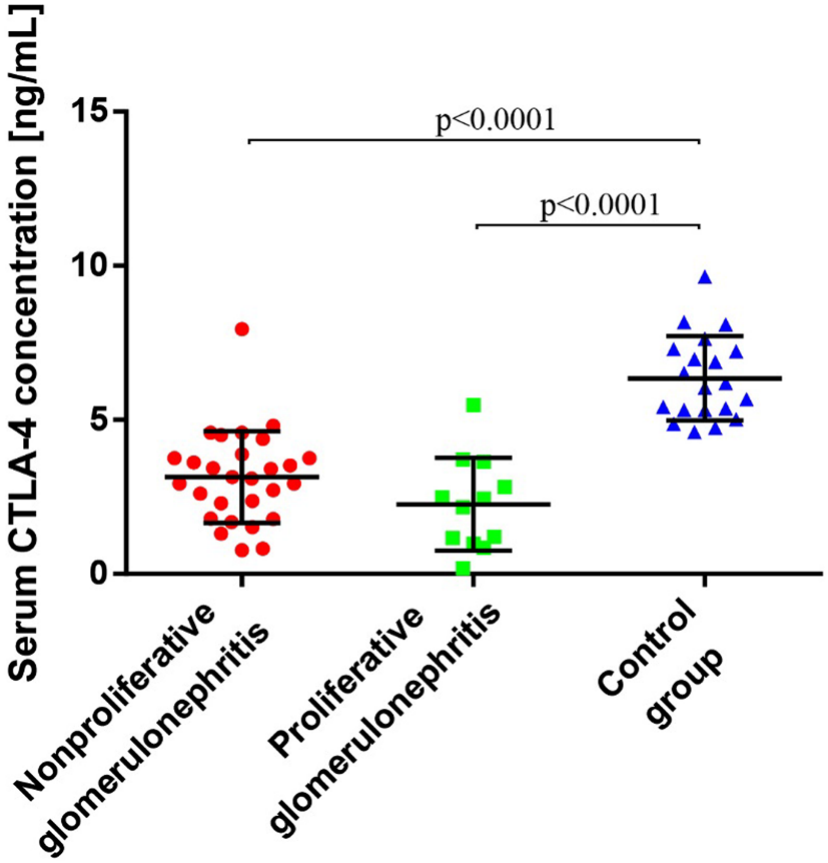
Serum sCTLA-4 concentration in patients with proliferative glomerulonephritis, patients with nonproliferative glomerulonephritis, and healthy volunteers

The concentration of sCTLA-4 was lower among patients with PGN [2.262 ± 1.511 ng/mL; median 2.303 ng/mL (0.179–5.485 ng/mL)] and NPGN [3.14 ± 1.489 ng/mL; median 3.12 ng/mL (0.769–7.94 ng/mL)] than in the control group [6.349 ± 1.364 ng/mL; median 6.12 ng/mL (4.603–9.648 ng/mL)] (Table 3; Fig. 2).

### ROC Curve Analysis to Determine the Diagnostic Accuracy of CTLA-4^+^ Lymphocytes and sCTLA-4 Concentration in Patients with NPGN or PGN vs Controls

**Fig. 3:**
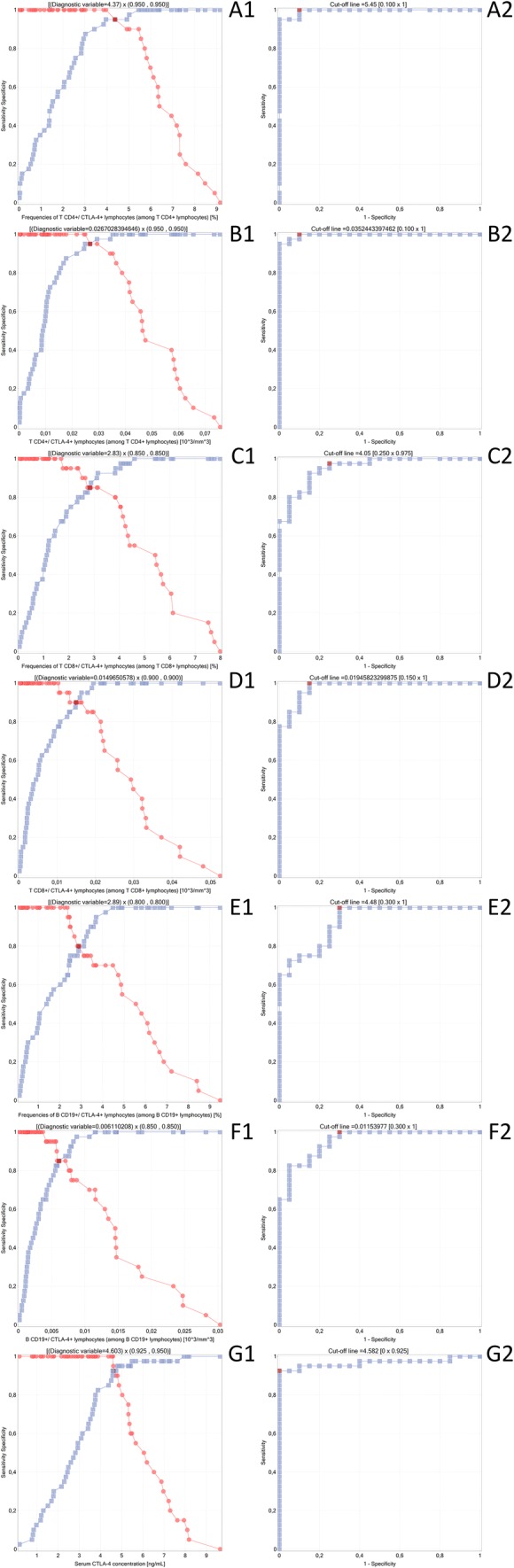
Receiver operating characteristic (ROC) curve to compare immunological parameter sensitivity and specificity in patients with nonproliferative glomerulonephritis and proliferative glomerulonephritis vs healthy controls: **a** frequencies of CD4^+^/CTLA-4^+^ T lymphocytes (among CD4^+^ T lymphocytes); **b** CD4^+^/CTLA-4^+^ T lymphocytes (among CD4^+^ T lymphocytes) (10^3^/mm^3^); **c** frequencies of CD8^+^/CTLA-4^+^ T lymphocytes (among CD8^+^ T lymphocytes) (%); **d** CD8^+^/CTLA-4^+^ T lymphocytes (among CD8^+^ T lymphocytes) (10^3^/mm^3^); **e** frequencies of CD19^+^/CTLA-4^+^ B lymphocytes (among CD19^+^ B lymphocytes) (%); **f** CD19^+^/CTLA-4^+^ B lymphocytes (among CD19^+^ B lymphocytes) (10^3^/mm^3^); **g** serum CTLA-4 concentration [ng/mL]

The results of the ROC curve analysis (Table 4; Fig. 3) revealed that all the analyzed parameters were highly sensitive and specific to distinguish patients with NPGN or with PGN from controls.

**Table 4:** ROC curve analysis to determine the diagnostic accuracy of immunological parameters in patients with NPGN and PGN versus healthy controls

Analyzed variables	Frequencies of CD4^+^/CTLA-4^+^ T lymphocytes (among CD4^+^ T lymphocytes) (%)—NPGN vs. PGN	CD4^+^/CTLA-4^+^ T lymphocytes (among CD4^+^ T lymphocytes) (10^3^/mm^3^)—NPGN vs. PGN	Frequencies of CD8^+^/CTLA-4^+^ T lymphocytes (among CD8^+^ T lymphocytes) (%)—NPGN vs. PGN	CD8^+^/CTLA-4^+^ T lymphocytes (among CD8^+^ T lymphocytes) (10^3^/mm^3^)—NPGN vs. PGN	Frequencies of CD19^+^/CTLA-4^+^ B lymphocytes (among CD19^+^ B lymphocytes) (%)—NPGN vs. PGN	CD19^+^/CTLA-4^+^ B lymphocytes (among CD19^+^ B lymphocytes) (10^3^/mm^3^)—NPGN vs. PGN	Serum CTLA-4 concentration [ng/mL]
AUC	0.995	0.99625	0.95375	0.97875	0.92875	0.95375	0.96625
SE(AUC)	0.004901	0.00389	0.024331	0.014932	0.032219	0.024553	0.023808
–95% CI	0.985394	0.988625	0.906063	0.949484	0.865602	0.905627	0.919586
+95% CI	1	1	1	1	0.991898	1	1
*Z* statistic	6.209775	6.225457	5.692294	6.005919	5.378669	5.692294	5.849107
*p* value	< 0.000001	< 0.000001	< 0.000001	< 0.000001	< 0.000001	< 0.000001	< 0.000001

*AUC* area under the curve, *CI* confidence interval, *NPGN* nonproliferative glomerulonephritis, *PGN* proliferative glomerulonephritis, *ROC* receiver operating characteristic

### CTLA-4^+^ T and B Lymphocytes Correlate Negatively with Selected Laboratory Parameters

**Fig. 4:**
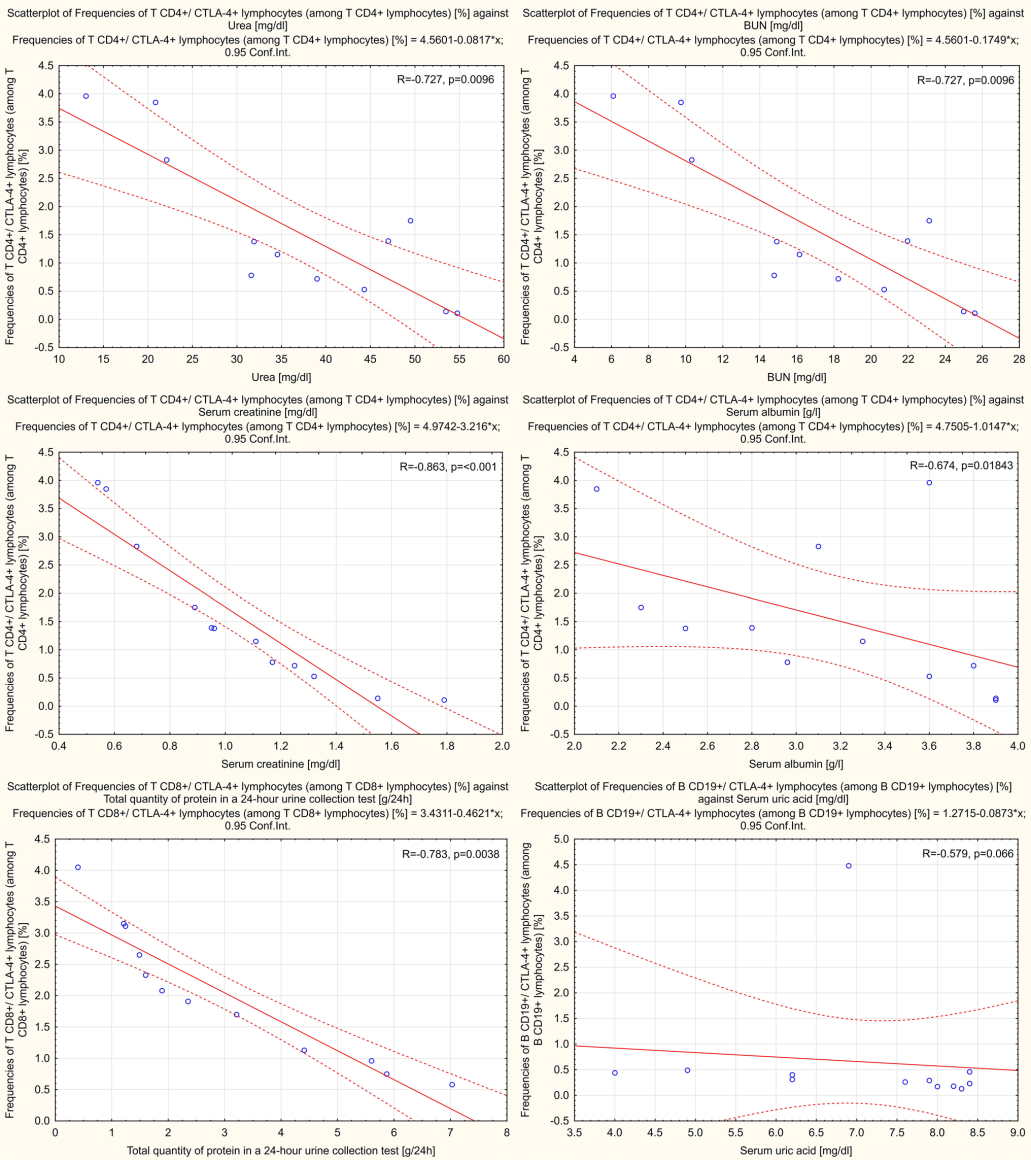
Frequency scatter plots of lymphocytes against selected clinical parameters for patients with proliferative glomerulonephritis

**Fig. 5:**
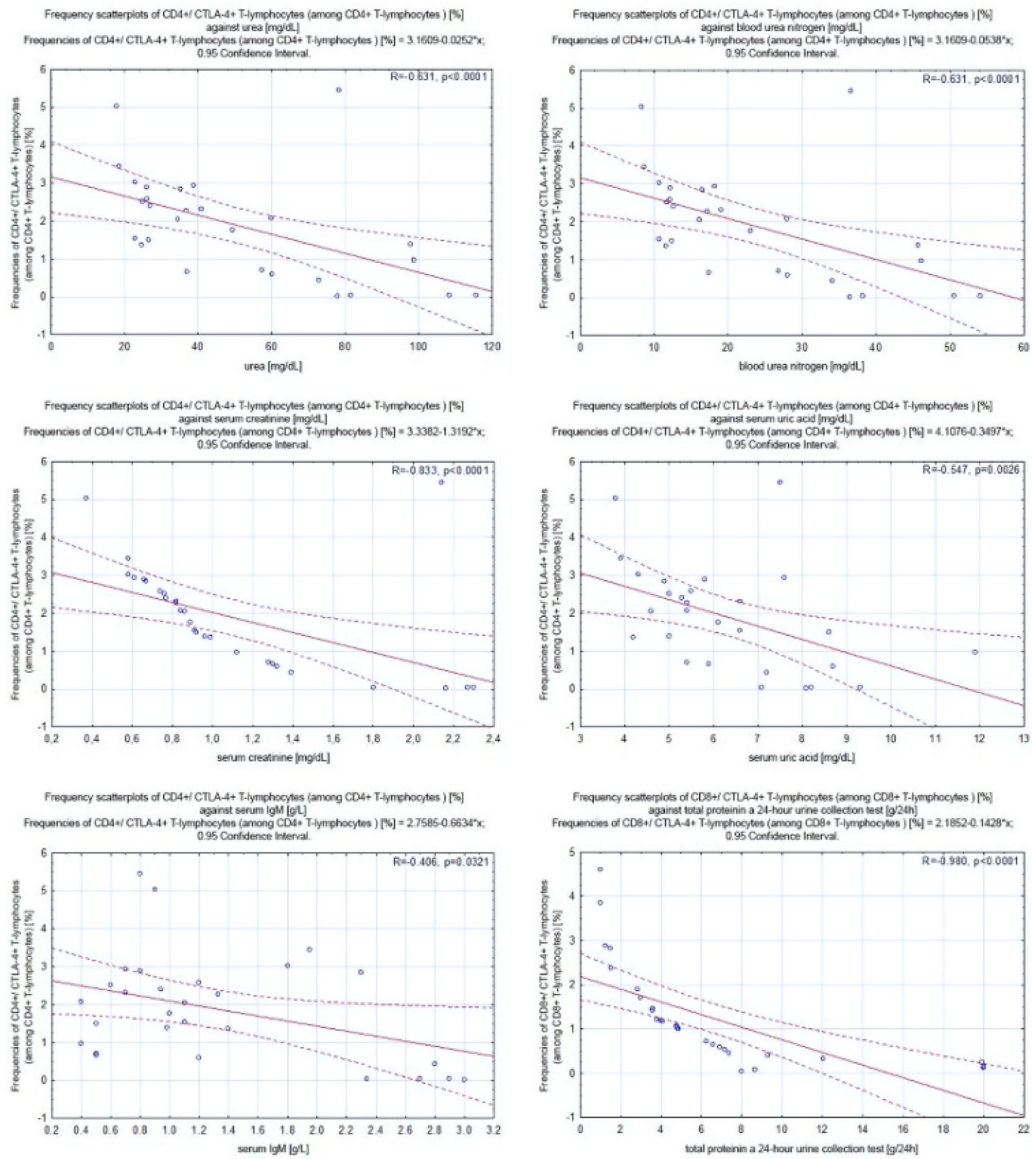

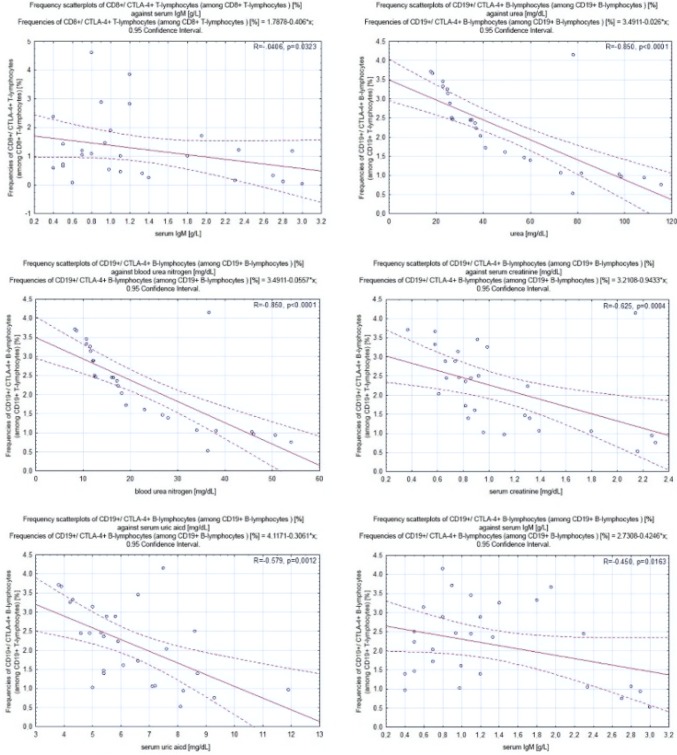
Frequency scatter plots of lymphocytes against selected clinical parameters for patients with nonproliferative glomerulonephritis

Frequency scatter plots of lymphocytes against selected clinical parameters for patients with PGN and NPGN are presented in Figs. 4 and 5, respectively.

#### Correlations in Patients with PGN

In patients with PGN, we found a negative correlation between the frequencies of CD4^+^/CTLA-4^+^ T lymphocytes and the concentration of urea (− 0.73; *p* = 0.0096), blood urea nitrogen (BUN) (− 0.73; *p* = 0.0096), serum creatinine (− 0.86; *p* < 0.001), and serum albumin (− 0.67; *p* = 0.01843). Moreover, we found a negative correlation between the frequencies of CD8^+^/CTLA-4^+^ T lymphocytes and the concentration of total protein in a 24-h urine collection test (− 0.78; *p* = 0.0038) and between the frequencies of CD19^+^/CTLA-4^+^ B lymphocytes and the concentration of serum uric acid (− 0.58; *p* = 0.066) (Fig. 4).

#### Correlations in Patients with NPGN

In patients with NPGN, we found a negative correlation between the frequencies of CD4^+^/CTLA-4^+^ T lymphocytes and the concentration of urea (− 0.63; *p* < 0.0001), BUN (− 0.63; *p* < 0.0001), serum creatinine (− 0.83; *p* < 0.0001), serum uric acid (− 0.55; *p* = 0.0026), and serum IgM (− 0.41; *p* = 0.0321). We also found a negative correlation between the frequencies of CD8^+^/CTLA-4^+^ T lymphocytes and the concentration of total protein in a 24-h urine collection test (− 0.98; *p* < 0.0001) and serum IgM (− 0.41; *p* = 0.0323). Moreover, we found a negative correlation between the frequencies of CD19^+^/CTLA-4^+^ B lymphocytes and the concentration of urea (− 0.85; *p* < 0.0001), BUN (−0.85; *p* < 0.0001), serum creatinine (− 0.63; *p* = 0.0004), serum uric acid (− 0.58; *p* = 0.0012), and serum IgM (− 0.45; *p* = 0.0163) (Fig. 5).

### Serum sCTLA-4 Concentration Correlates Negatively with Selected Laboratory Parameters

**Fig. 6:**
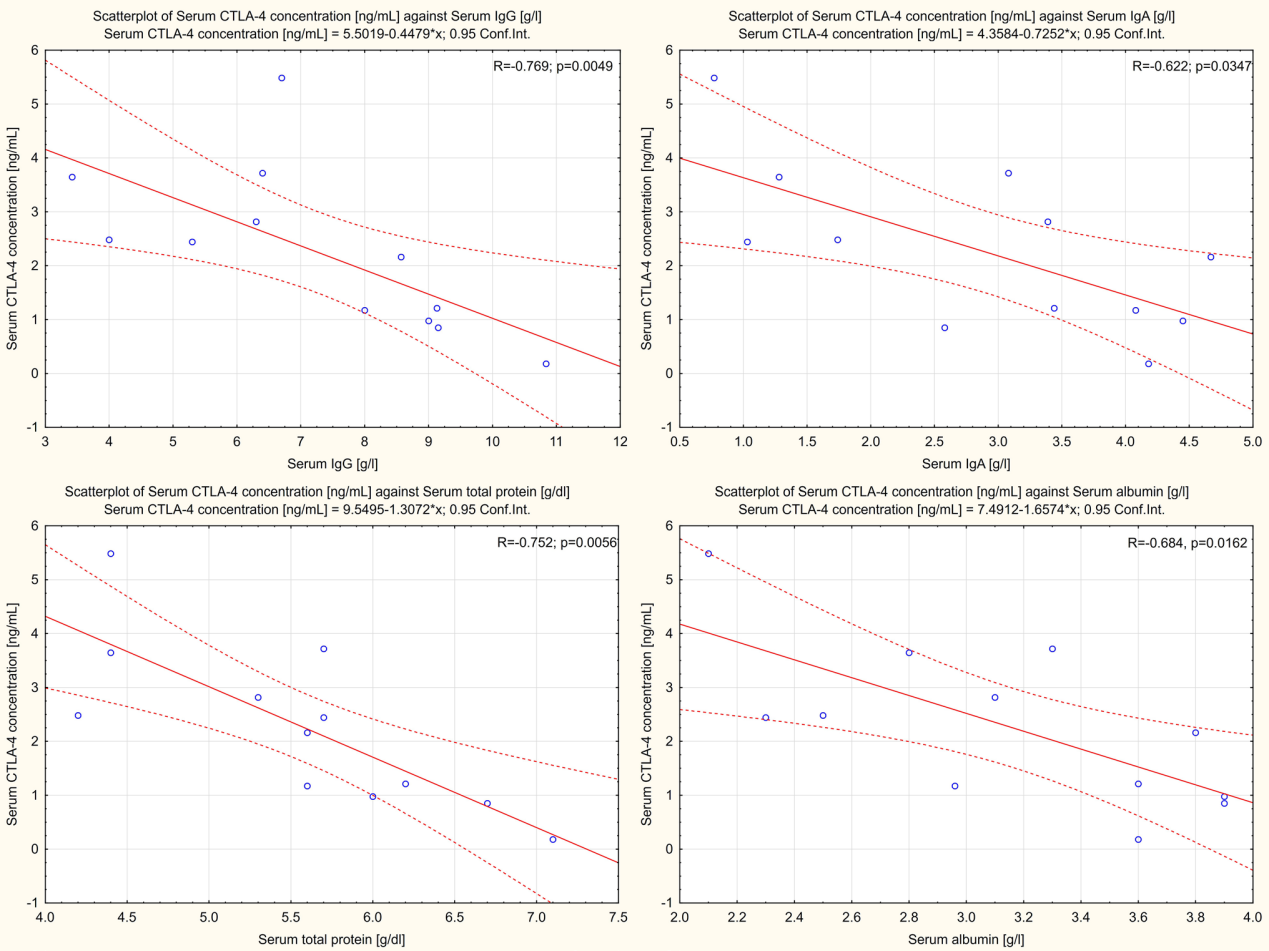
Concentration scatter plots of sCTLA-4 against selected clinical parameters for patients with proliferative glomerulonephritis

We found a negative correlation between the serum concentration of sCTLA-4 and the serum concentrations of IgG (− 0.77; *p* = 0.0049), IgA (− 0.62; *p* = 0.0347), total protein (− 0.75; *p* = 0.0056), and albumin (− 0.68; *p* = 0.0162) (Fig. 6).

## Discussion

CTLA-4 is a T-cell membrane receptor that plays a key role in regulating T-cell activation and the maintenance of immune homeostasis. CTLA-4 binds the same ligands as CD28 (B7-1 and B7-2), but in contrast to CD28, acts as a negative regulator of T-cell activation (Hoff et al. 2009; Krummel and Allison 1995). In addition to its multiple mechanisms of T-cell inhibition, CTLA-4 function is further expanded by the existence of three alternatively spliced isoforms: sCTLA-4, liCTLA-4, and 1/4CTLA-4. However, the role of CLTA-4 in the progression of chronic renal disease remains elusive.

The purpose of this study was to evaluate the role of CTLA-4 in the pathogenesis of primary GN. We found that the expression of CTLA-4 on the cell membrane of T and B cells, as well as the serum sCTLA-4 concentration, was lower in patients with PGN and NPGN than in controls. The lower expression of CTLA-4 on the membranes of T cells translates into a higher number of activated T cells and a higher concentration of inflammatory cytokines. Low percentages of lymphocytes with CTLA-4 expression may contribute to constant T-cell activation and the development of glomerular inflammation and injury. These results suggest a direct involvement of CTLA-4 in the pathogenesis of PGN and NPGN.

Many SNPs associated with autoimmune diseases have been identified for the CTLA-4 gene (Ueda et al. 2003). For example, for the −318T/T and +49AA genotype, an increased expression of cell-surface CTLA-4 was detected and was associated with a reduced susceptibility to autoimmune diseases (Carr et al. 2009; Ligers et al. 2001). On the other hand, the +49GG genotype, associated with decreased expression of CTLA-4 (Maurer et al. 2002), increases the risk of MCD, focal segmental glomerulosclerosis, and membranous nephropathy. This indicates that CTLA-4 plays a role in the pathogenesis of primary nephrotic kidney diseases (Spink et al. 2013). Decreased expression of CTLA-4 has also been found in tonsillar T cells in patients with palmoplantar pustulosis (Harabuchi 2011). Additionally, the +49GG genotype was more common in patients with renal involvement in pediatric Henoch–Schönlein purpura (Wang et al. 2012), and the CT60 polymorphism was associated with a decreased risk of Graves’ disease (Du et al. 2013) and an increased susceptibility to rheumatoid arthritis (Lei et al. 2005).

Many studies suggest that the SNPs of CTLA-4 are associated with the pathogenesis of IgA nephropathy. An abnormal expression of the CTLA-4 gene has been connected with an increased risk of several autoimmune diseases, like rheumatoid arthritis, infectious diseases, transplantation, and asthma (Carreno and Collins 2002; Keir and Sharpe 2005; Khoury and Sayegh 2004). CTLA-4 gene polymorphisms are present in patients with IgA nephropathy, the most common autoimmune kidney disease worldwide (D’Amico 1987; Wyatt and Julian 2013). Furthermore, it was shown that these SNPs are involved in the development of this disease (Gorgi et al. 2010). The CTLA-4 rs231726 gene is associated with a higher risk of developing IgA nephropathy (Wang et al. 2014), and the CTLA-4 rs231779 gene is associated with proteinuria, podocyte foot process effacement, and advanced mesangial proliferation (Kim et al. 2011). Additionally, data obtained by Jacob et al. (2018) support the hypothesis that CTLA-4 may be involved in the development and progression of IgA nephropathy. They found that frequencies of the –318/C/T SNP within the CTLA-4 gene locus appreciably differ between IgA nephropathy patients and healthy controls. Moreover, the presence of the SNPs +49A/G and CT60 G/A within the CTLA-4 gene locus were associated with higher proteinuria (> 1 g/day) in patients with IgA nephropathy.

A lower concentration of sCTLA-4 in patients with PGN and NPGN than in controls can contribute to constant T-cell activation, leading to the development of glomerular inflammation and injury. CTLA-4 is decreased in the serum and the urine of patients with MCD (Garin et al. 2010). Other studies have shown that urine concentrations of sCTLA-4 in patients with relapsed MCD were not statistically different compared with those in remission. However, the ratio of sCD80/CTLA-4 in urine was > 100-fold higher in patients with relapsed MCD compared with those in remission. In contrast, concentrations of soluble CD80 and CTLA-4 in serum did not differ among patients with MCD in relapse and in remission (Garin et al. 2009).

Due to the correlation of CD80 and CD86 expression in many glomerulopathies, the B7-1(CD80)-CTLA-4 and B7-2(CD86)-CTLA-4 interactions have been proposed as a molecular target in the treatment of these diseases. Currently, a blockade of immune checkpoints is considered the most effective immunotherapy approach. The development of antibodies, known as immune checkpoint inhibitors, contributed considerably to the improvement of patient outcomes in several malignancies. CTLA-4 and programmed cell death protein 1 (PD-1) are two essential immune checkpoint receptors (Rusch et al. 2018). The US Food and Drug Administration has approved ipilimumab and tremelimumab (anti-CTLA-4-blocking antibodies) and pembrolizumab and nivolumab (antibodies targeting PD-1 receptors), currently under clinical investigation (Izzedine et al. 2017). Abatacept, the extracellular ligand-binding domain of CTLA-4 bound to the Fc portion of IgG1, binds to B7-1 by blocking CD28 or potentiating CTLA-4 signals. As a result, abatacept may be a specific tool to decrease proteinuria (Trimarchi 2015) in B7-1-positive podocytes. However, the efficacy of abatacept in treating focal segmental glomerulosclerosis is controversial (Garin et al. 2015; Yu et al. 2013). Deregulation of T-cell inhibitory receptor PD-1 may contribute to PGN and NPGN pathogenesis (Grywalska et al. 2018).

ROC curve analysis showed the clinical utility of the seven immunological parameters related to CTLA-4. The percentages and absolute counts of CD4^+^/CTLA-4^+^, CD8^+^/CTLA-4^+^, and CD19^+^/CTLA-4^+^ cells and the serum sCTLA-4 concentration were sensitive and specific parameters to characterize patients with PGN and NPGN. These proteins can be, therefore, considered molecular biomarkers and valuable diagnostic tools in patients with PGN and NPGN (Caster et al. 2018).

Moreover, in patients with NPGN, we found a statistically significant negative correlation between the concentration of serum IgM and the frequencies of CD4^+^/CTLA-4^+^ T lymphocytes, CD8^+^/CTLA-4^+^ T lymphocytes, and CD19^+^/CTLA-4^+^ B lymphocytes. Most serum IgM is produced spontaneously by a distinct subset of B cells and reacts with a variety of epitopes expressed on both self- and non-self-antigens. Secreted IgM molecules remove altered self-antigens such as apoptotic cells and are crucial in the induction of B-cell central tolerance (Nguyen and Baumgarth 2016). Induced by CTLA-4 isoforms, the inhibition of the immune response may be associated with inappropriate IgM function. As a result, NPGN patients experience a loss of B-cell central tolerance, an accumulation of altered self-antigens, and glomerular injury. Other studies have shown that the concentration of serum IgM in patients with NPGN was significantly positively correlated with the frequencies of CD4^+^/PD-1^+^ and CD4^+^/PD-L1^+^ T cells (Grywalska et al. 2018). An analysis of the relationships between laboratory parameters and the serum concentration of sCTLA-4 revealed a significant negative correlation between sCTLA-4 and the serum concentrations of IgG, IgA, total protein, and albumin.

In conclusion, our results shed new light on the role of the CTLA-4 isoforms in the pathogenesis of PGN and NPGN. Low frequencies of lymphocytes expressing CTLA-4 and low serum concentration of sCTLA-4 may contribute to continuous T-cell activation and the development of glomerular inflammation and injury. Future studies evaluating CTLA-4 expression levels in affected kidneys will help to understand the role of CTLA-4 in primary GN and its potential as a therapeutic target.

Because our results were obtained from a relatively small study group, further research needs to be done. We did not evaluate the patients’ CTLA-4^+^ lymphocytes and sCTLA-4 in kidney biopsies. Our preliminary data are promising, but they require confirmation and extension by describing the CTLA-4-dependent pathway in patients treated with medications that affect the immune system. A description of the correlations between overall survival and the changes in CTLA-4 expression is also missing. Further research should include an assessment of CTLA-4 expression on higher cell populations, including cells in glomeruli and glomerular cell infiltrates. The expression of CTLA-4 during the treatment of GN, as well as after remission, should also be evaluated.
